# Gene expression profiling of host lipid metabolism in SARS-CoV-2 infected patients: a systematic review and integrated bioinformatics analysis

**DOI:** 10.1186/s12879-024-08983-0

**Published:** 2024-01-23

**Authors:** Wan Amirul Syazwan Wan Ahmad Munawar, Marjanu Hikmah Elias, Faizul Helmi Addnan, Pouya Hassandarvish, Sazaly AbuBakar, Nuruliza Roslan

**Affiliations:** 1 Faculty of Medicine and Health Sciences, Universiti Sains Islam Malaysia, Nilai, Malaysia; 2 Tropical Infectious Diseases Research and Education Centre (TIDREC), Universiti Malaya, Kuala Lumpur, Malaysia

**Keywords:** SARS-CoV-2, Lipid metabolism, Gene expression, Bioinformatics, Next-generation sequencing

## Abstract

**Background:**

The Coronavirus disease 2019 (COVID-19) pandemic occurred due to the dispersion of severe acute respiratory syndrome coronavirus 2 (SARS-CoV-2). Severe symptoms can be observed in COVID-19 patients with lipid-related comorbidities such as obesity and diabetes. Yet, the extensive molecular mechanisms of how SARS-CoV-2 causes dysregulation of lipid metabolism remain unknown.

**Methods:**

Here, an advanced search of articles was conducted using PubMed, Scopus, EBSCOhost, and Web of Science databases using terms from Medical Subject Heading (MeSH) like SARS-CoV-2, lipid metabolism and transcriptomic as the keywords. From 428 retrieved studies, only clinical studies using next-generation sequencing as a gene expression method in COVID-19 patients were accepted. Study design, study population, sample type, the method for gene expression and differentially expressed genes (DEGs) were extracted from the five included studies. The DEGs obtained from the studies were pooled and analyzed using the bioinformatics software package, DAVID, to determine the enriched pathways. The DEGs involved in lipid metabolic pathways were selected and further analyzed using STRING and Cytoscape through visualization by protein-protein interaction (PPI) network complex.

**Results:**

The analysis identified nine remarkable clusters from the PPI complex, where cluster 1 showed the highest molecular interaction score. Three potential candidate genes (*PPARG*, *IFITM3* and *APOBEC3G*) were pointed out from the integrated bioinformatics analysis in this systematic review and were chosen due to their significant role in regulating lipid metabolism. These candidate genes were significantly involved in enriched lipid metabolic pathways, mainly in regulating lipid homeostasis affecting the pathogenicity of SARS-CoV-2, specifically in mechanisms of viral entry and viral replication in COVID-19 patients.

**Conclusions:**

Taken together, our findings in this systematic review highlight the affected lipid-metabolic pathways along with the affected genes upon SARS-CoV-2 invasion, which could be a potential target for new therapeutic strategies study in the future.

**Supplementary Information:**

The online version contains supplementary material available at 10.1186/s12879-024-08983-0.

## Introduction

The novel severe acute respiratory syndrome coronavirus 2 (SARS-CoV-2) is the pathogen responsible for the Coronavirus Disease 2019 (COVID-19) pandemic. The virus comprises an enveloped single-stranded, positive-sense RNA and it belongs to the *Betacoronavirus* genus of the subfamily *Orthocoronavirinae* in the *Coronaviridae* family [1]. SARS-CoV-2 has a 79% common sequence identity with SARS-CoV-1, which caused the Asian SARS outbreak from 2002 to 2004 [2]. COVID-19 was first reported in Wuhan, China, where the patient was hospitalized on December 12th, 2019 [3]. Since then, the pandemic has infected 634 million people worldwide and caused around 6.6 million deaths up to November 2022, (WHO, 2022). Before the introduction of vaccines, the pandemic was considered a fatal threat to humanity. With the rapid rise in cases and no available cure, many healthcare systems worldwide were burdened, prompting governments to impose lockdowns in a bid to stem the infection.

However, with the introduction of vaccines and anti-viral medications like Paxlovid, the disease has become manageable and less fatal. It is now commonly characterized by systemic inflammation [4], with mild to severe fever and coughs, besides shortness of breath and chest pain. However, patients with comorbidities, such as cardiovascular disease, diabetes, obesity and cancer, tend to develop severe consequences [5]. This may happen due to the dysregulation of genes responsible for various signaling pathways associated with the comorbidities, such as the immune response and cell growth. The alteration of the genes involved may subsequently lead to the enhancement of SARS-CoV-2 pathogenicity.

Recently, several studies have focused on the association between lipid metabolic pathways and the pathogenicity of SARS-CoV-2 because patients with co-morbidities tended to develop severe symptoms of COVID-19. A study by Al Heialy et al. (2020) found that obese and diabetic people were more likely to be afflicted with severe pulmonary inflammation and injury [6]. This observation is strengthened by the fact that obesity may lower the effectiveness of the immune response towards infection or vaccination [7]. It is known that obesity and diabetes are highly associated with the dysregulation of lipid synthesis and clearance [6]. Wang et al. (2021) proposed that the identification of host transcriptional response to SARS-CoV-2 infection be divided into two components, namely material metabolism and cytokine-related transcriptional regulation [8]. Dysregulation of lipid metabolism may increase the expression of angiotensin converting enzyme 2 (ACE2), which was suggested by Al Heialy et al. (2020) based on in silico and in vitro findings. ACE2 is expressed in various tissues, such as the lungs, kidney, heart, gallbladder, liver and intestines, and is usually bound to the cell membrane, although some may exist in soluble form in the blood [9]. This enzyme plays an important role in the renin-angiotensin-aldosterone system (RAAS) to control blood pressure in humans. However, membrane-bound ACE2 has also been identified as the binding site for SARS-CoV-2 infection. Therefore, patients with lipid dysregulation will subsequently be at risk of severe SARS-CoV-2 infection due to their increased expression of ACE2.

A multi-omics study can analyze changes in host transcriptomic profiling before and after COVID-19 infection [10]. At the transcriptomic level, many studies on clinical samples of SARS-CoV-2 patients have generated enormous numbers of DEGs [11–15]. However, there is still no systematic reviews or in silico analyses of DEGs from COVID-19 patients to determine changes in molecular mechanisms related to lipid metabolism. Therefore, this study aims to identify the significant DEGs from previous studies and execute a bioinformatics analysis to identify the enriched lipid metabolic pathways that may facilitate or enhance viral pathogenicity. This systematic review and integrated bioinformatics analysis will provide an insight into molecular mechanisms involved in SARS-CoV-2 infection, specifically those involving lipid metabolism-related pathways.

## Methods

This review had been officially listed in PROSPERO (No. CRD42022336734).

### Search strategy

This article search was systematically performed according to the Preferred Reporting Items for Systematic Reviews and Meta-Analyses (PRISMA) guidelines. An extensive literature search on gene expression profiling of SARS-CoV-2 and host metabolism was conducted on PubMed, Scopus, EBSCOhost and Web of Science electronic databases, and all articles published until July 3, 2022, were collected. The searching method involved the use of Medical Subject Heading (MeSH) terms from NCBI and Boolean operators, which were as follows: (“SARS-CoV-2” OR “2019-nCoV” OR “COVID-19” OR “2019 Novel Coronavirus” OR “Coronavirus Disease 2019” OR “Severe Acute Respiratory Syndrome Coronavirus 2” OR “Coronavirus Disease-19” OR “SARS Coronavirus 2”) AND (“Lipid Metabolism” OR “Lipogenesis” OR “Lipolysis” OR “Lipid” OR “Fatty Acid Metabolism” OR “Triglyceride Metabolism” OR “Triacylglycerol Metabolism” OR “Cholesterol Metabolism” OR “Phospholipid Metabolism” OR “Sphingolipid Metabolism” OR “Eicosanoids Metabolism” OR “Cholesterol”) AND (“Gene Expression” OR “Gene Expression Regulation” OR “Transcription” OR “Transcriptome” OR “Transcriptomes” OR “Transcriptomic” OR “Transcriptional”). The term “Transcriptional” had been included in the literature search, which was obtained through the evaluation of relevant papers. Additional papers were picked out from the references of the collected studies.

### Inclusion criteria

Gene expression profiling or transcriptomic studies analyzing DEGs of individuals infected with SARS-CoV-2 were included. In addition, only clinical studies using RNA-sequencing (next-generation sequencing) to analyze DEGs in COVID-19 patients were selected to ensure the accuracy and uniformity of reported outcomes. For single-cell RNA-seq data, the DEGs data were analyzed using related software (e.g., MAST in Seurat v.3) to ensure the removal of data’s heterogeneity, make them comparable to standard RNA-seq data. Lastly, datasets of DEGs with absolute log fold change > 1 and *p*-value of < 0.1 were selected for further analysis.

### Exclusion criteria

Studies without original data, such as case reports, editorials, conference proceedings and review articles were rejected. Other exclusion criteria were studies on genomics, proteomics and metabolomics, studies without a healthy control group, and in vitro, in silico and in vivo studies. This review is anchored on the outcome of DEGs between COVID-19 patients and healthy individuals. Therefore, any studies implementing treatment or intervention, and those comparing DEGs between severities of infected patients were omitted. These criteria were used as selection guidelines for achieving the aim of this systematic review in analyzing the significant studies on gene expression of infected SARS-CoV-2 individuals, which facilitated the determination of dysregulated genes and pathways involved in infection.

### Articles’ screening for acceptability

Article papers acquired from databases and other sources were screened in three stages. First, duplicates were removed and all articles having titles and abstracts that did not fulfill the inclusion criteria were not retrieved. Finally, the full texts of the retrieved studies were examined in-depth. All articles that did not meet the inclusion criteria and had any one of the exclusion criteria were excluded. All authors were engaged in screening and selecting the retrieved articles.

### Data extraction

Data from the selected studies were extracted with the involvement of all authors to discuss differences in opinion. The following data were included: (A) title and author’s name, (B) study design, (C) study objective, (D) study population, (E) type of sample used, (F) method used in gene expression analysis, (G) number of DEGs and (H) conclusion.

### Study quality assessment

All authors examined and reviewed the quality of the selected studies independently. The assessment was based on Joanna Briggs Institute critical tools (https://jbi.global/critical-appraisal-tools) [16], according to the type of study. The exclusion of biases was done by attaching to the inclusion criteria. The quality assessment results were validated by discussion and consensus among reviewers.

### Differentially expressed genes (DEGs) and functional annotation analysis

The DEGs were pooled from selected studies. The replicates of the DEGs were removed and the common DEGs between at least three studies were selected for further analysis. Next, the DEGs identified were analyzed using the Database for Annotation, Visualization, and Integrated Discovery (DAVID) software (https://david.ncifcrf.gov/tools.jsp) [17, 18]. The analysis via DAVID was done to identify the set of genes displaying significant functional annotation during infection by SARS-CoV-2. The genes’ involvement in the pathways enriched in SARS-CoV-2 infected patients were determined according to the Kyoto Encyclopedia of Genes and Genomes (KEGG) pathway, Biological Biochemical Image Database (BBID), BIOCARTA pathway database and Reactome. The terms acquired from the analysis were filtered by selecting terms with a *p*-value of < 0.05. Next, only lipid-related terms, which involved the dysregulation of host lipid metabolism upon SARS-CoV-2 infection, were selected through discussion.

### Protein-protein interaction complex and clustering

The collected DEGs involved in lipid-related terms by DAVID were then analyzed at the protein level to identify the protein-protein interaction complex based on their related enriched pathways using the STRING (PPI Functional enrichment analysis) software (https://string-db.org/) [19]. The data from STRING were then transferred to the Cytoscape bioinformatics software (http://www.cytoscape.org/) to visualize the molecular interaction complexes and incorporate gene expression profiles [20]. The Molecular Complex Detection (MCODE) plug-in function in Cytoscape was used to execute the module analysis of targeted network and clustering of proteins [21]. The module-selection criteria included degree cut-off of 2 for network scoring, node score cut-off of 0.2, node density cut-off of 0.1, K-score of 2, and maximum depth of 100 for cluster finding. The genes involved in each cluster were then analyzed separately in DAVID to determine the remarkable enriched ontology terms.

## Results

### Eligible studies selected according to PRISMA guidelines

**Fig. 1:**
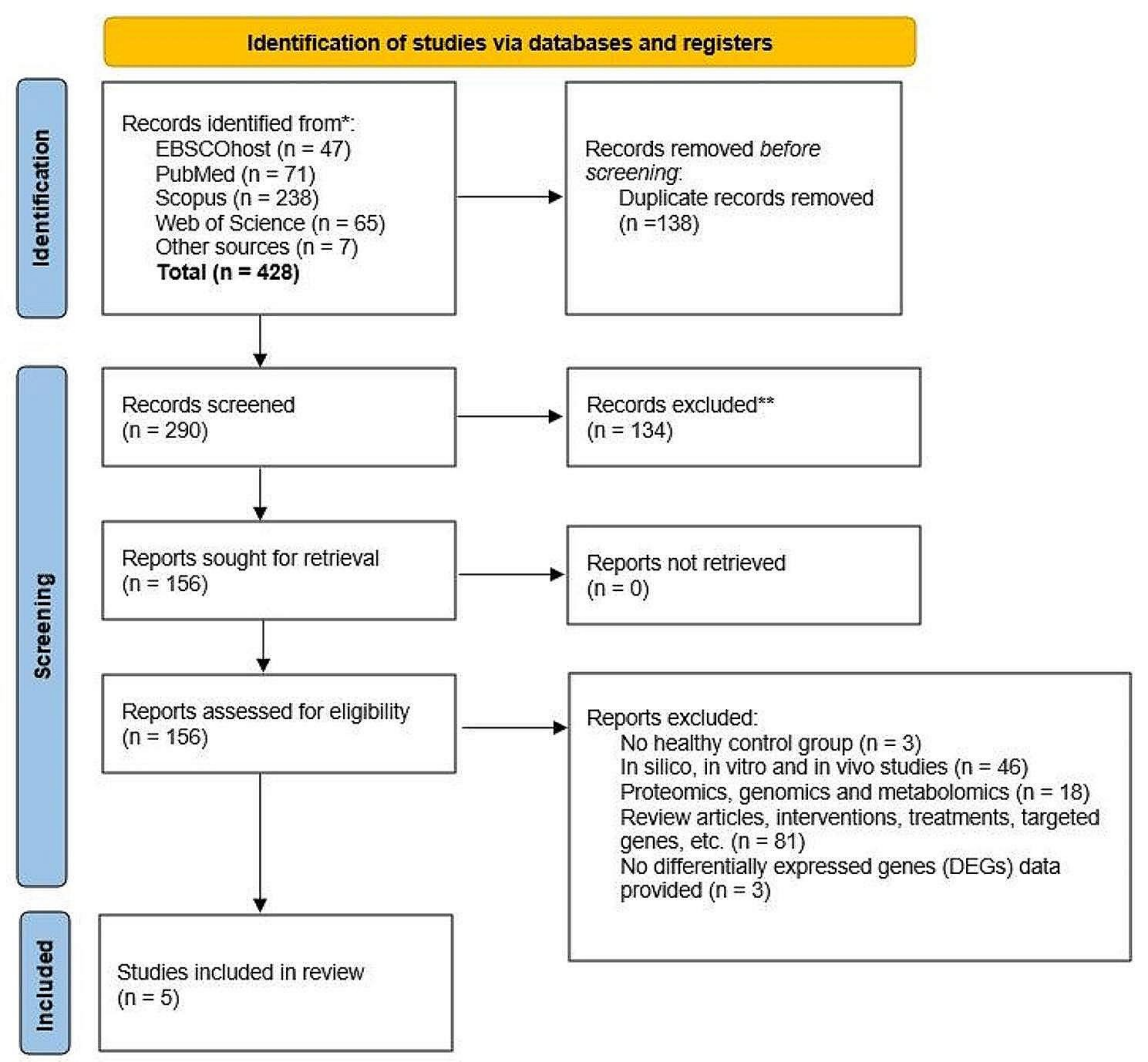
Flow diagram for selection of studies according to PRISMA guidelines

The literature search produced 421 articles from the four databases (EBSCOhost, PubMed, Scopus, and Web of Science) and another seven from related sources. In the filtering process, 138 articles were identified as duplicates. Based on the titles and abstracts, the first screening stage found that 134 articles were not related to the study and therefore, were removed. The second stage of the screening process was performed by reviewing the full texts of the remaining 156 articles, and after applying the inclusion and exclusion criteria, had resulted in the elimination of 151 articles. The final five articles were selected for systematic review. The flow diagram of the screening process and reasons for the articles’ exclusion are shown in Fig. 1.

### Quality of selected studies

All selected studies have a low-risk bias scoring 70 to 100% (high quality). The details of the studies’ quality assessment are shown in S1 Table.

### Characteristics of selected studies

The selected studies were primary research articles published from 2020 to 2022. The uniformity of the selected studies was assured by applying the inclusion and exclusion criteria to avoid bias. All selected articles were case-control studies that used RNA-seq Next Generation Sequencing (NGS) to analyze gene expression. The population size in each study ranged from 2 to 430 subjects, with a total of 565 individuals involved, including controls. The characteristics of these studies were summarized in Table 1.

**Table 1:** Summary of selected studies

Title (References)	Authors (year)	Study Population	Sample type	Method for gene expression analysis	No. of DEGs
Imbalanced Host Response to SARS-CoV-2 Drives Development of COVID-19 [11]	Blanco-Melo et al. (2020)	COVID-19 human (*n* = 2) Uninfected human (*n* = 2)	Lung tissues	RNA-seq analysis TruSeq (Illumina)	23,710
Transcriptional profiling of leukocytes in critically ill COVID19 patients: implications for interferon response and coagulation [12]	Gill et al. (2020)	COVID-19 + ICU patients (*n* = 7) COVID-19- ICU patients (*n* = 7)	Buffy coat cells from blood (Leukocytes)	RNA-seq (Illumina NextSeq 500)	1311
Single-cell landscape of bronchoalveolar immune cells in patients with COVID-19 [13]	Liao et al. (2020)	COVID-19 patients (*n* = 13) Healthy controls (*n* = 3)	Bronchoalveolar lavage fluids (BALFs)	single-cell RNA-seq (scRNA-seq)	2874
In vivo antiviral host transcriptional response to SARS-CoV-2 by viral load, sex, and age [14]	Lieberman et al. (2020)	PCR-confirmed SARS-CoV-2 (*n* = 430) Negative controls (*n* = 54)	Nasopharyngeal swabs	Metagenomic next-generation sequencing (mNGS) @ RNA-seq (Illumina NextSeq or Illumina NovaSeq)	83
Time-resolved systems immunology reveals a late juncture linked to fatal COVID-19 [15]	Liu et al. (2021),	Hospitalized COVID-19 patients (*n* = 33) Healthy controls (*n* = 14)	Peripheral blood mononuclear cells (PBMCs)	Cellular Indexing of Transcriptomes and Epitopes by Sequencing (CITE-seq)	6187

### Identification of DEGs in COVID-19 patients

Blanco-Melo et al. (2020) provided the highest number of DEGs, with 23,710 genes having the expression of absolute log2 fold change > 1 and *p*-value of < 0.05, obtained by comparing lung biopsies from COVID-19 patients with healthy lung tissue from uninfected individuals, who were all males aged above 60. Gill et al. (2020) and Lieberman et al. (2020) had provided 1.311 and 83 DEGs, respectively. Gill et al. (2020) selected only genes with an expression level of more than absolute 1.5-fold change and false discovery rate (FDR) step-up *p*-value cut-off of ≤ 0.0545. As for Lieberman et al. (2020), the inclusion criteria were an absolute log2 fold change of > 1 and p-adjusted value of < 0.1. The study by Gill et al. (2020) collected blood samples from COVID-19 patients upon admission into the intensive care unit (ICU), while Lieberman et al. (2020) used nasopharyngeal (NP) swabs from infected individuals confirmed through RT-PCR and negative controls as well.

**Fig. 2:**
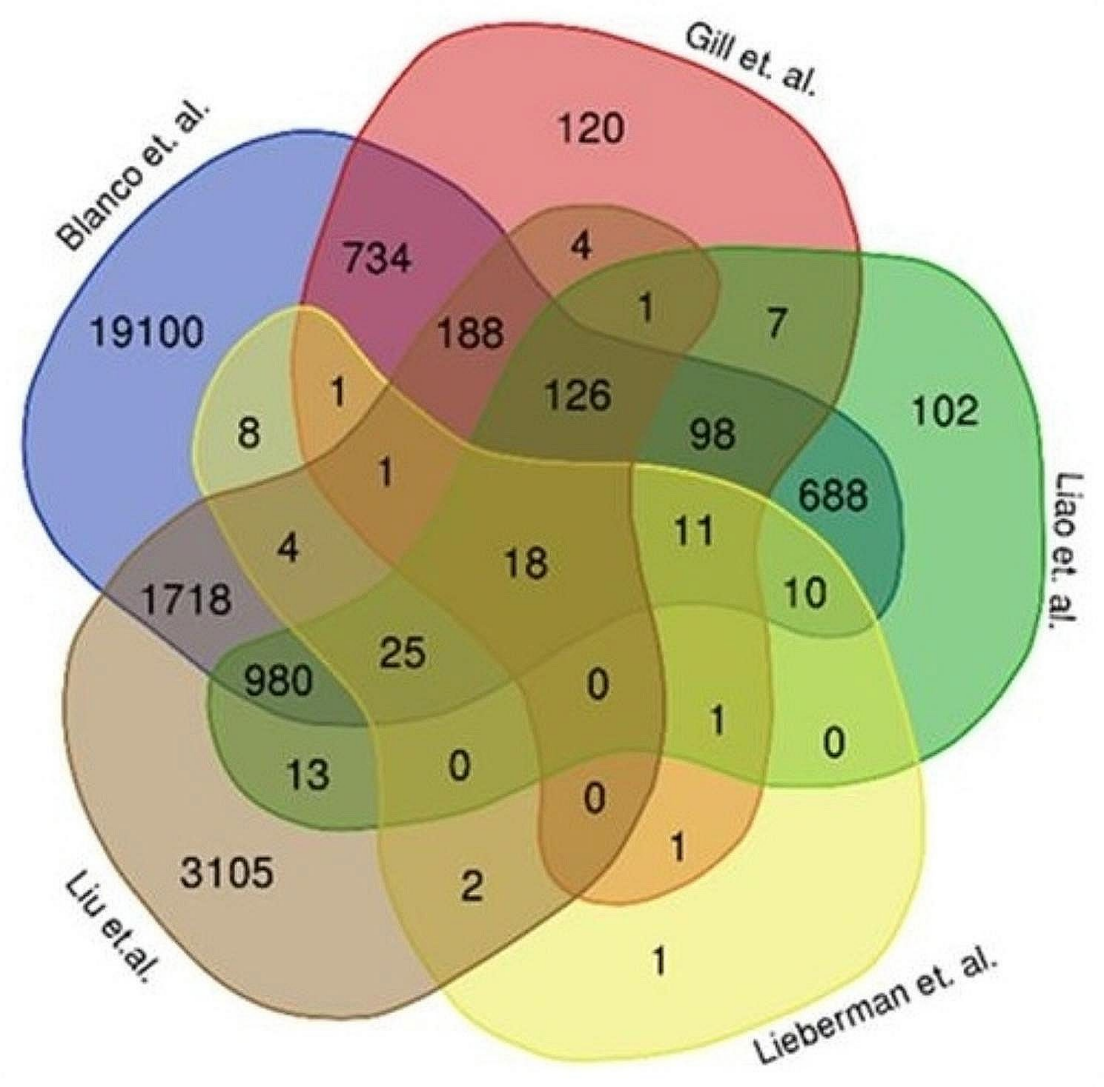
Distribution of DEGs among the five selected studies. Each study is represented in different colors. The overlapping areas indicate the common DEGs

As for Liu et al. (2021), the Cellular Indexing of Transcriptomes and Epitopes by Sequencing (CITE-seq) was done on peripheral blood mononuclear cells (PBMCs) from hospitalized COVID-19 patients and healthy controls with matched age and gender. A total of 6187 DEGs were identified under their selection criteria; log-fold change greater than 0.25, expressed in at least 10% of the PBMC samples and *p*-value of < 0.01. Meanwhile, in Liao et al. (2020), scRNA-seq was performed on bronchoalveolar lavage fluid (BALF) cells from moderate and severe COVID-19 patients and healthy controls. The DEGs from macrophage subclusters and T lymphocyte cluster were further analyzed, which resulted in the discovery of 1547 and 1327 DEGs with adjusted *p*-values of < 0.05, respectively. The distribution of DEGs among five studies is summarized in Fig. 2. The DEGs were analyzed by selecting those that were common in at least three studies. As a result, 1464 DEGs were identified.

### Functional annotation of DEGs and selection of lipid metabolism-related DEGs

A total of 1464 DEGs were analyzed to determine the genes’ functional annotation by Gene Ontology (GO) analysis using DAVID. The full record of remarkable functional annotations of DEGs common in at least three studies is provided in S2 Table. Then, the terms related to lipid metabolism were selected and further analyzed. The lipid-related terms were selected through discussion among the authors. The DEGs from lipid-related terms were then extrapolated. As a result, 213 DEGs were identified to be involved in lipid metabolisms-related terms.

The pathways/terms identified were categorized into three databases; Uniprot (UP), KEGG pathway, and GO term enrichment analysis (GOTERM). Most DEGs were involved in the lipoprotein’s (KW-0449) post-translational modification (UP_KW_PTM). This finding is parallel with functional annotation of biological process (BP) by GO that includes the cellular response to low-density lipoprotein particle stimulus, lipoprotein transport and cholesterol regulation. The record of functional annotations of the genes related to lipid metabolisms terms is summarized in Table 2.

**Table 2:** Functional annotation of the DEGs related to lipid metabolisms terms

Term	Description	Count	*p*-value
has05417	Lipid and atherosclerosis	54	8.18E-09
hsa04932	Non-alcoholic fatty liver disease	41	1.53E-07
GO:0043548	phosphatidylinositol 3-kinase binding	7	0.006106
GO:0071404	cellular response to low-density lipoprotein particle stimulus	9	7.05E-05
GO:0019216	regulation of lipid metabolic process	8	0.024861
GO:0042953	lipoprotein transport	6	0.010306
GO:0070542	response to fatty acid	6	0.027794
GO:0010875	positive regulation of cholesterol efflux	6	0.044065
GO:0010888	negative regulation of lipid storage	5	0.004233
GO:0032367	intracellular cholesterol transport	5	0.012068
GO:0010887	negative regulation of cholesterol storage	4	0.041673
KW-0449	Lipoprotein	118	3.34E-05
KW-0564	Palmitate	52	0.001456

### Potential DEGs and their terms in protein-protein interaction (PPI) complex

All 213 DEGs identified to be involved in lipid metabolism pathways were analyzed using the STRING online database. The list of 213 DEGs is provided in S3 Table. As a result, 213 proteins were refined into a protein-protein interaction complex, displaying 210 nodes and 1929 edges with a PPI enrichment *p*-value of < 1.0e-16.

**Fig. 3:**
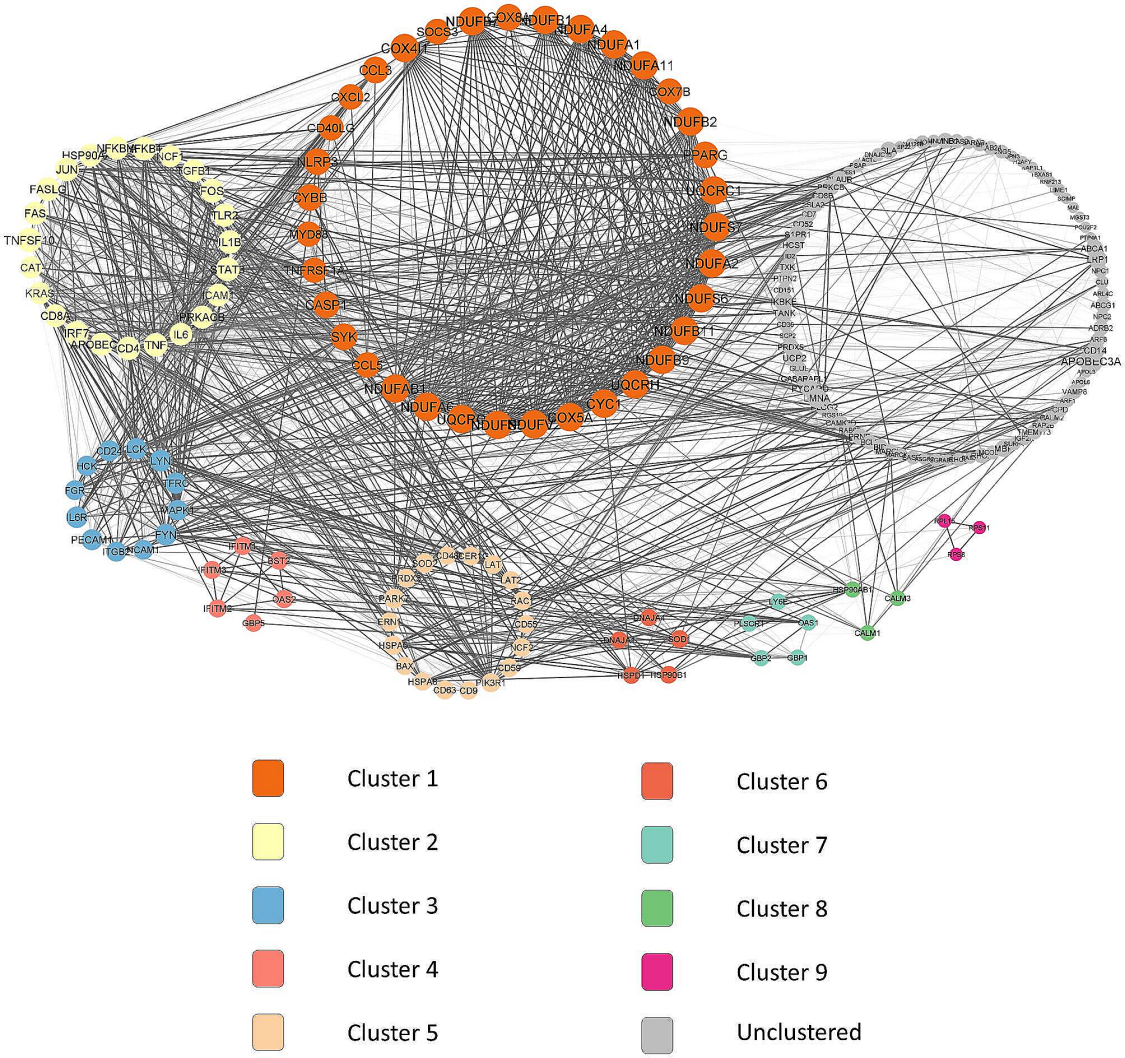
PPI complex and modular analysis of selected DEGs. A total of 198 proteins were refined into a PPI complex from STRING online databases analysis. Cytoscape MCODE plug-in identified nine clusters from the DEGs

The STRING outcome data were exported to Cytoscape to provide a vision on the molecular interaction networks. Nine remarkable clusters from the PPI network complex were identified using the Cytoscape MCODE plug-in. Figure 3 shows the PPI complex results from the DEGs involved in human lipid metabolism-related terms upon SARS-CoV-2 infection.

Functional annotation clustering revealed that cluster 1 (score = 17.765) encompassed 35 nodes and 302 edges. Most of the DEGs in cluster 1 were located in the mitochondria, which were involved in aerobic respiration and protein binding, and associated with non-alcoholic fatty liver disease. Cluster 2 (score = 16.091) comprised 23 nodes and 177 edges. The locations of the DEGs in this cluster were the plasma membrane and cytosol. Most of the DEGs were associated with transcription regulation, as well as contributors to lipid dysfunction and atherosclerotic pathways.

Cluster 3 (score = 6.909) comprised 12 nodes and 38 edges, mostly in the plasma membrane, which were associated with immunity and host-virus interaction. Cluster 4 (score = 5.2), comprised of six nodes and 13 edges, and was linked to responses towards virus infection. Cluster 5 (score = 4.235), comprising 18 nodes and 36 edges, was associated with protein binding and negative regulation of apoptosis.

Clusters 6 and 7 (score = 4, respectively) comprised five nodes and eight edges. Cluster 6 was associated with chaperone binding, while cluster 7 was highly associated with lipoprotein. The last two clusters, cluster 8 and cluster 9 (score = 3 each), each shared three nodes and three edges, respectively. Cluster 8 was involved in the estrogen signaling pathway, while cluster 9 was associated with ribosomal protein functions and translation. The list of DEGs according to their cluster is shown in Table 3.

**Table 3:** Clustering details of DEGs involved in lipid metabolism terms in SARS-CoV-2-infected individuals

Cluster	Score	Nodes	Edges	DEGs
1	17.765	35	302	*COX7B, NDUFA11, NDUFA1, SYK, NDUFA4, NDUFB1, SOCS3, NDUFAB1, COX8A, NDUFA6, UQCRQ, MYD88, NDUFS5, NDUFV2, TNFRSF1A, COX5A, CYC1, NDUFB7, UQCRH, NDUFB9, NDUFB11, NDUFS6, COX4I1, NDUFA2, NDUFS7, CCL3, UQCRC1, CXCL2, CASP1, CD40LG, NLRP3, CYBB, NDUFB2, CCL5, PPARγ*
2	16.091	23	177	*JUN, TGFB1, FASLG, IRF7, ICAM1, STAT3, FAS, APOBEC3G, IL1B, TLR2, TNFSF10, CAT, CD4, NCF1, KRAS, TNF, NFKB1, IL6, NFKBIA, HSP90AA1, PRKACB, FOS, CD8A*
3	6.909	12	38	*TFRC, LYN, LCK, MAPK1, CD24, NCAM1, FYN, IL6R, ITGB2, HCK, PECAM1, FGR*
4	5.2	6	13	*GBP5, IFITM3, IFITM2, OAS2, IFITM1, BST2*
5	4.235	18	36	*CD63, FCER1G, HSPA5, CD48, RAC1, CD9, SOD2, LAT2, BAX, PRDX3, PARK7, HSPA8, PIK3R1, CD59, LAT, NCF2, ERN1, CD55*
6	4	5	8	*DNAJA4, SOD1, HSP90B1, HSPD1, DNAJA1*
7	4	5	8	*OAS1, GBP1, PLSCR1, LY6E, GBP2*
8	3	3	3	*HSP90AB1, CALM3, CALM1*
9	3	3	3	*RPS11, RPL15, RPS8*

The record of remarkable functional annotations of all DEGs in their corresponding clusters is shown in Table 4. The full record of remarkable functional annotations of all DEGs in their corresponding clusters is provided in S4 Table.

**Table 4:** Functional annotation clustering of each cluster determined from the DEGs

Cluster	Term	Description	Count	*p*-value
1	CC_GO:0005743	mitochondrial inner membrane	23	1.61E-28
	CC_GO:0005739	mitochondrion	19	1.07E-12
	CC_GO:0005747	mitochondrial respiratory chain complex I	15	4.57E-29
	MF_GO:0005515	protein binding	28	0.041873
	BP_GO:0009060	aerobic respiration	16	8.63E-29
	BP_GO:0042776	mitochondrial ATP synthesis coupled proton transport	14	1.74E-24
	BP_GO:0006120	mitochondrial electron transport, NADH to ubiquinone	13	4.93E-24
	hsa04932	Non-alcoholic fatty liver disease	26	5.56E-37
2	CC_GO:0005886	plasma membrane	16	2.05E-05
	CC_GO:0005829	cytosol	14	0.001414
	BP_GO:0045893	positive regulation of transcription, DNA-templated	11	2.25E-09
	BP_GO:0045944	positive regulation of transcription from RNA polymerase II promoter	11	2.78E-07
	MF_GO:0042802	identical protein binding	15	3.93E-10
	hsa05417	Lipid and atherosclerosis	17	1.97E-21
3	hsa04650	Natural killer cell mediated cytotoxicity	4	0.000541
	hsa04062	Chemokine signaling pathway	4	0.001838
	hsa04660	T cell receptor signaling pathway	3	0.008202
	hsa04659	Th17 cell differentiation	3	0.008822
4	CC_GO:0016020	membrane	4	0.015637
	BP_GO:0045071	negative regulation of viral genome replication	5	1.41E-10
	BP_GO:0009615	response to virus	5	5.15E-09
	BP_GO:0051607	defense response to virus	5	1.02E-07
	KW-0391	Immunity	6	3.32E-06
	KW-0051	Antiviral defense	5	9.92E-08
5	BP_GO:0043066	negative regulation of apoptotic process	5	0.000977
	KW-0564	Palmitate	7	3.33E-06
	KW-0945	Host-virus interaction	5	0.002533
6	MF_GO:0051087	chaperone binding	4	7.68E-07
	KW-0143	Chaperone	4	2.77E-05
7	KW-0449	Lipoprotein	5	1.8E-05
8	BP_GO:0071902	positive regulation of protein serine/threonine kinase activity	3	1.05E-05
	hsa04915	Estrogen signaling pathway	3	0.000284
9	CC_GO:0022626	cytosolic ribosome	3	1.38E-05
	CC_GO:0005840	ribosome	3	7.29E-05
	BP_GO:0002181	cytoplasmic translation	3	2.15E-05
	BP_GO:0006412	translation	3	0.000133
	MF_GO:0003735	structural constituent of ribosome	3	9.98E-05

Based on the PPI network, three potential candidate genes had been chosen for further analysis, which were peroxisome proliferator-activated receptor gamma (*PPARγ*), apolipoprotein B mRNA editing enzyme catalytic subunit 3G (*APOBEC3G*) and interferon-induced transmembrane protein 3 (*IFITM3*). Those genes were chosen based on their protein functions, which significantly regulated lipid metabolism. Based on the MCODE algorithm, the scores for the candidate genes were as follows; *PPARγ* = 18, *APOBEC3G* = 14 and *IFITM3* = 8. The details on the functions and terms related to candidate genes are provided in S5 Table.

**Fig. 4:**
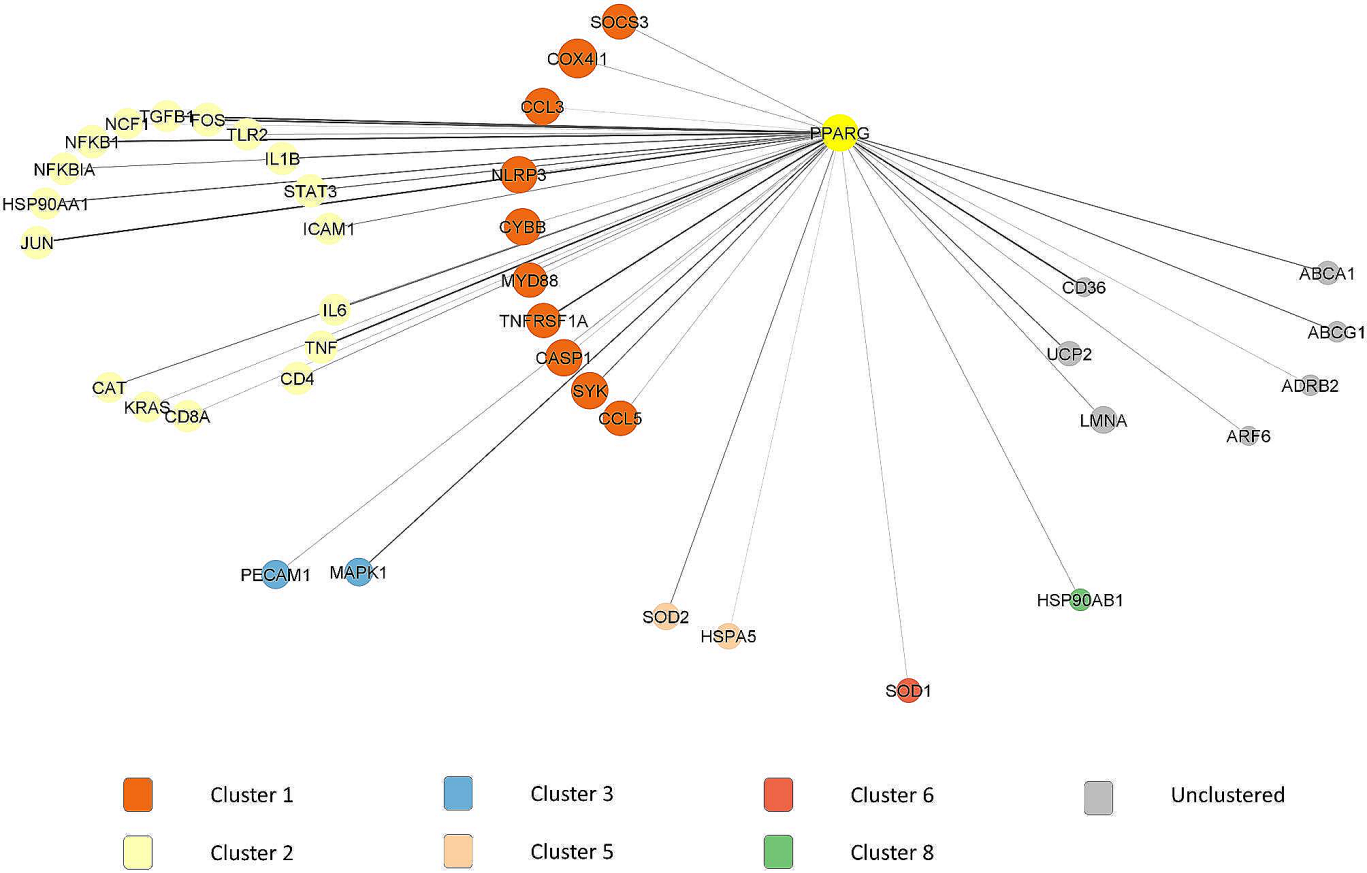
First DEGs neighbors of *PPARγ* in PPI network and modular analysis

*PPARγ* (score = 18) was highly interconnected with other genes in the same and other clusters, which were cluster 2, cluster 3, cluster 5, cluster 6, and cluster 8. It had the greatest number of interactions with cluster 2 (17 out of 23 DEGs). The highest interaction of *PPARγ* in this cluster was with *JUN* (combined score = 0.984), followed by *TNF* (combined score = 0.979). The *PPARγ* interactions with other DEGs are shown in Fig. 4.

**Fig. 5:**
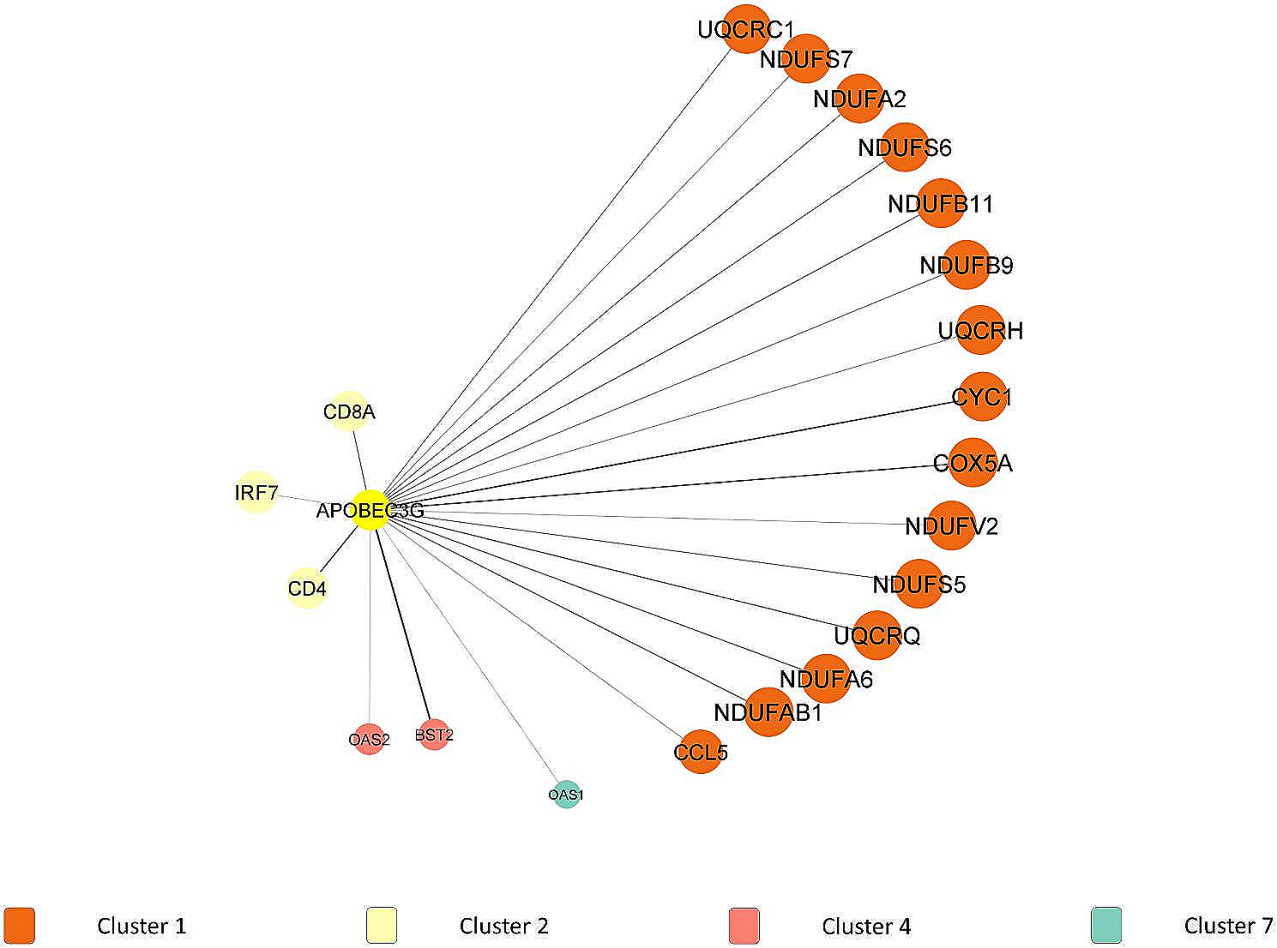
First DEGs neighbors of *APOBEC3G* in PPI network and modular analysis

*APOBEC3G* was highly interacted with cluster 1 (15 out of 35 DEGs), followed by cluster 4 and cluster 7. The highest interaction of *APOBEC3G* could be seen with *BST2* (combined score = 0.88) in cluster 4, followed by interaction with *CYC1* (combined score = 0.772) in cluster 1, as shown in Fig. 5.

**Fig. 6:**
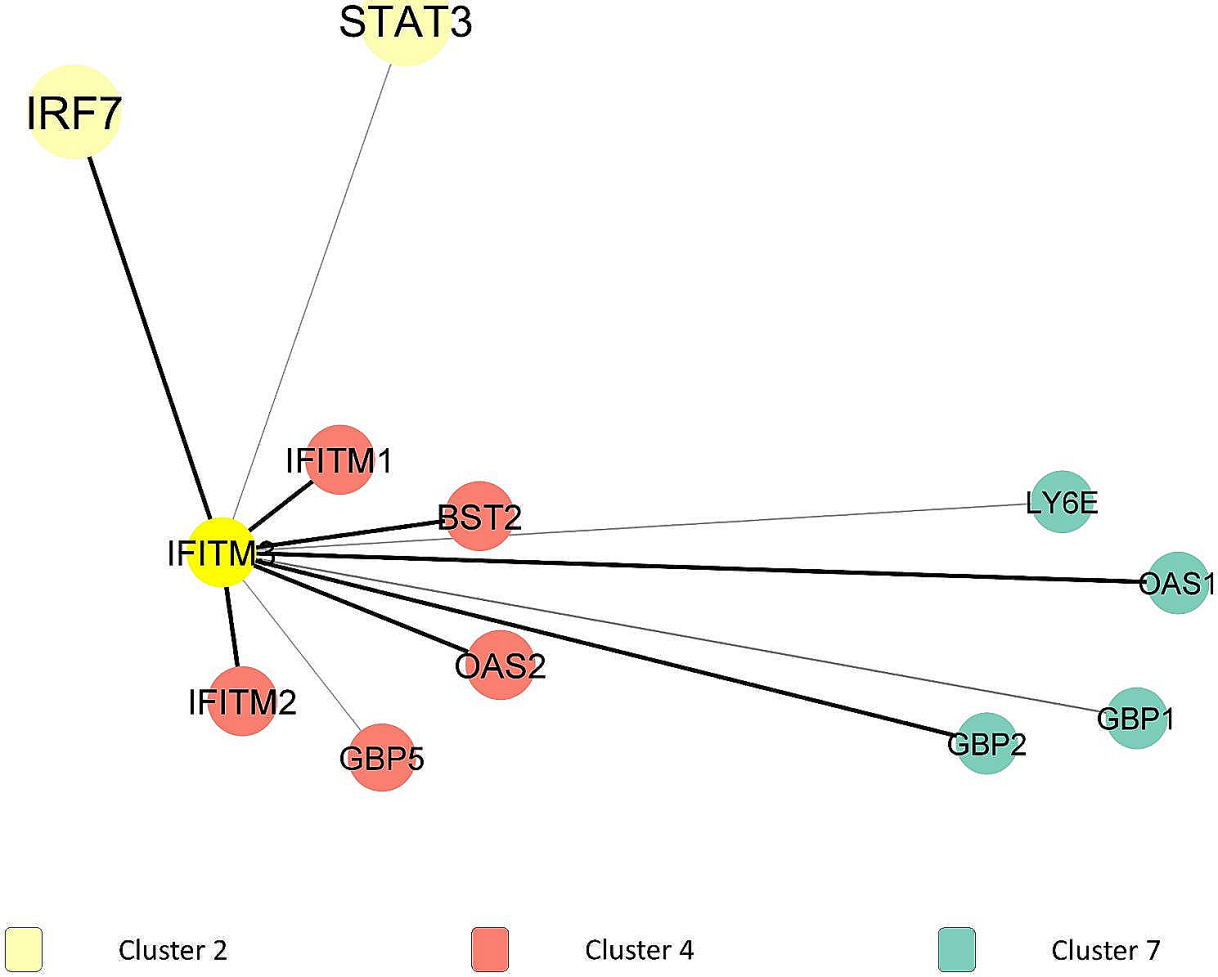
First DEGs neighbors of *IFITM3* in PPI network and modular analysis

*IFITM3* was interconnected with all DEGs in the same cluster (cluster 4), which was *IFITM1*, *BST2*, *OAS2*, *GBP5*, and *IFITM2*, where the highest interaction could be seen between *IFITM3* and *IFITM2* (combined score = 0.988), followed by *IFITM3* and *IFITM1* (combined score = 0.973). Plus, *IFITM3* also had interactions with cluster 2 (*IRF7* and *STAT3*) and 80% of DEGs from cluster 7, which were *LY6E*, *OAS1*, *GBP1*, and *GBP2*. The interactions held by *IFITM3* could be seen in Fig. 6. All the data regarding the edges (combined score of DEG interactions) are provided in S6 Table.

## Discussion

The comprehension of dysregulated genes during infection by SARS-CoV-2 was indispensable to determine the potential pathways involved during viral entry into the host cell, in this case, lipid metabolism-related pathways. Based on this systematic review, several clinical studies had investigated the changes in gene expression of SARS-CoV-2-infected patients [11–15]. Therefore, through integrated bioinformatics analysis, the DEGs provided by those studies were analyzed for their contribution in altering the host lipid metabolism during infection. The analysis results in the identification of nine clusters that were interconnected in a network complex, namely the PPI network. Three potential candidate genes were identified from the PPI network, which were *PPARγ*, *APOBEC3G* and *IFITM3*. These genes were selected based on their significant function in regulating host lipid metabolism.

The *PPARγ* protein is a nuclear receptor that binds peroxisome proliferators like fatty acids. Once a ligand activates this receptor, it will bind to DNA-specific PPAR-response elements. *PPARγ* is a crucial regulator for adipocyte differentiation and glucose homeostasis. In addition, it is also a transcription factor that coordinates the expression of genes related to reproduction, metabolism and immune response. Due to their anti-inflammatory properties, *PPARγ* ligands had been proposed as anti-SARS-CoV-2 drugs [22]. *PPARγ* was arranged in cluster 1, where it was found to be involved in several terms, such as in innate immune response (GO:0045087), negative regulation of inflammatory response (GO:0050728) and cellular response to low-density lipoprotein particle stimulus (GO:0071404). *PPARγ* is responsible for regulating lipid metabolism and adipogenesis [23–25], where it controls the genes involved in the release, transport and storage of fatty acids, such as the fatty acid transporter CD36 [25]. Many diseases had been linked to the dysregulation of *PPARγ*, such as obesity, type 2 diabetes and atherosclerosis [25]. The gene seemed to interact with *TNF*, which might be related to the suppression of *PPARγ* expression by *TNF-a* [26]. *PPARγ* dysregulation can also be strengthened by the metabolomic and proteomic study analyzing the serum of COVID-19 patients by Yang et al. (2021), who reported that differential metabolites obtained were responsible for the PPAR signaling pathway and differentially expressed proteins (DEPs) were involved in NF-kappa B signaling pathway, respectively [27]. The interplay between *PPARγ* and NF-kappa B is one of the immune responses’ critical regulators, via the antagonizing ability of *PPARγ* towards NF-kappa B [28].

*PPARγ* had the greatest number of interactions with cluster 2. DAVID analysis of cluster 2 revealed that several terms were related to lipid metabolism, as *PPARγ* also regulated lipid metabolism. The terms involved were response to insulin (GO:0032868), negative regulation of lipid storage (GO:0010888), regulation of insulin secretion (GO:0050796), negative regulation of fat cell differentiation (GO:0045599), sequestering of triglyceride (GO:0030730), insulin resistance (hsa04931), adipocytokine signaling pathway (hsa04920) and lipoprotein (KW-0449). These DEGs in cluster 2 were highly responsible for lipid-related disorders, such as diabetes and obesity, where insulin resistance occurs, and differentiation of adipocytes is disrupted. This result was in parallel with the function of *PPARγ* as a vital regulator of adipocyte differentiation and glucose homeostasis. Not to mention, some of the terms in cluster 2 were also associated with pathogenicity and replication of SARS-CoV-2. Some of the related terms were inflammatory response (GO:0006954), defense response to virus (GO:0051607), and positive regulation of cell division (GO:0051781). These terms were also related to *PPARγ*-associated terms in cluster 1, which was the innate immune response and inflammatory response, where they were activated upon virus infection. Plus, disruption in cell division and proliferation regulation might indicate that these genes in cluster 2 were responsible for viral replication in the host. Therefore, further study on the relationship of *PPARγ* and DEGs in cluster 2 with association towards viral replication should be taken into consideration to get insight regarding viral replication at the transcriptomic level.

*APOBEC3G* encodes for apolipoprotein B mRNA editing enzyme, catalytic subunit 3G. *APOBEC3G* is a member of the cytidine deaminase gene family. The protein encoded by this gene catalyzes site-specific deamination of both RNA and single-stranded DNA, inducing the conversion of cytosine to uracil [29]. This protein had been observed to act as an inhibitor of retrovirus replication through hypermutations, as well as other APOBEC3s, which were involved in restricting infection of viruses and propagation affecting viruses [30]. Currently, the SARS-CoV-2 genomic variations from analysis of databases presented a high C-to-U mutation rate, accounting for about two out of five single nucleotide variations, which was assumed to be the consequences of RNA editing by host APOBECs instead of mutations at random. Plus, the involvement of several APOBECs in gene editing of the SARS-CoV-2 genome was revealed where *APOBEC3G* shows the highest C > U editing rate at motif CC > CU compared to other APOBECs, which contributes to the viral mutation [29].

*APOBEC3G* is located in cluster 2, where it is involved in the following terms; defense response to virus (GO:0051607), protein binding (GO:0005515), identical protein binding (GO:0042802), cytosol (GO:0005829), host-virus interaction (KW-0945), human immunodeficiency virus 1 infection (hsa05170), Ubl conjugation (KW-0832) and lipoprotein (KW-0449). Interestingly, *APOBEC3G* was the first APOBEC known to be involved in antiviral immunity through its activity against HIV [30]. The binding of *APOBEC3G* with RNA has contributed towards its packaging during virus encapsidation [31]. Previously, a study had discovered a novel interrelationship between *APOBEC3G* raft association and virus encapsidation. A total of nine *APOBEC3G* derivations were analyzed, which resulted in all packaging-competent *APOBEC3G* derivations being related to lipid rafts, while all packaging-incompetent *APOBEC3G* derivations were unable to do so [31]. This viral encapsidation was necessary for *APOBEC3G* to confer its antiviral activity on the replication of progeny virions in the target cells [32]. *APOBEC3G*-mediated editing also contributed to the activation of effectors of adaptive immunity, which was CD8 + cytotoxic T cells (CTLs) [30]. This evidence explained the interaction of *APOBEC3G* with *CD8A* shown in the PPI network. Therefore, further studies on analyzing the involvement of *APOBEC3G* in regulating lipid-metabolic pathways, specifically upon SARS-CoV-2 infection, should be initiated since the details of its involvement are still unclear.

*IFITM3* is a gene that encodes for Interferon (IFN) induced transmembrane protein 3. Increased *IFITM3* expression was a regular feature of severe COVID-19 cases, which was reported in a study by Regino-Zamarripa et al. (2022) [33]. *IFITM3* is an IFN-induced antiviral protein that could cause havoc in the homeostasis of intracellular cholesterol. The disruption of cholesterol homeostasis was part of a mechanism to inhibit the entry of the COVID-19 virus by preventing its fusion with cholesterol-depleted endosomes. Therefore, this response would restrict cellular entry by many viral pathogens, such as Ebola virus and SARS-CoV-2 [34]. *IFITM3* interacted with all DEGs in the same cluster. The terms lysosome (KW-0458), endosome (KW-0967), response to virus (GO:0009615), defense response to virus (GO:00516057), lysosomal membrane (GO:0005765) and late endosome membrane (GO:0031902) contributed to virion degradation by the lysosome. *IFITM3* is concentrated in endo-lysosomal membranes [35] since IFITM proteins are one of the host factors that restrict virus infection by impeding with cellular entry at endosomes [33]. Furthermore, the mechanisms involved blocking membrane fusion pore formation by *IFITM3* in late endosomes [33, 36]. The viral particles would be retained in late endosomes, which would then be targeted for lysosomal degradation [37].

Next, lipoprotein (KW-0449) and negative regulation of viral entry into host cell (GO:0046597) were associated with disruption of cholesterol trafficking. As mentioned, previously, *IFITM3* was shown to agitate trafficking of cholesterol. A study had shown that the amphipathic helix of *IFITM3* could make alterations on lipid membranes in vitro in a cholesterol-dependent manner. Cholesterol could regulate the access of the enveloped virus into the cell since it was a vital regulator of the biomechanical properties of lipid bilayers. *IFITM3* disrupted the protein-regulating transportation function of cholesterol between the endoplasmic reticulum and late endosomes/multivesicular bodies, known as VAMP-associated Protein A (VAPA). The disruption of the protein resulting in *IFITM3* would trigger the accretion of cholesterol within late endosomes [36]. Another piece of evidence to support the disruption of cholesterol trafficking was the interaction between *IFITM3* and 80% of DEGs in cluster 7, where they were involved in lipoprotein (KW-0449). Plus, changes in the concentration of lipoprotein metabolites were also associated with COVID-19 severity as assessed by Chen et al. (2020). Most of the high-density lipoprotein (HDL) subclasses were observed to significantly drop from mild to severe patients when compared to healthy control while many of the low-density lipoprotein (LDL) subclasses were elevated from mild to severe patients [38]. These findings had proven that *IFITM3* played an essential role in degrading the viral particles through lysosomes and increased the membrane rigidity to prevent entry of SARS-CoV-2 in the host cell. Therefore, an investigation on utilizing *IFITM3* as another therapeutic target for SARS-CoV-2 infection should be further studied.

### Limitations

One of the limiting factors in this review is the refining of papers obtained from database searching due to different types of study, various methods, and statistical approaches applied by the studies. Moreover, the patient’s demographic profiles, such as age and comorbidities, followed by the type of clinical samples, might contribute to biasness of the retrieved DEGs. However, data homogeneity was could be maintained by strictly adhering to the inclusion criteria and selecting shared DEGs between retrieved studies. Plus, the data bias could be avoided by selecting next-generation sequencing results only for further analyses. Applying bioinformatics analyses would also help avoid bias due to human error, since the tools used were computational-based. Therefore, further in vitro, in vivo and clinical studies of *PPARγ*, *IFITM3* and *APOBEC3G* genes were needed to decipher the genes’ involvement in regulating lipid metabolic pathways and viral pathogenicity as predicted through these in silico analyses. Currently, an experimental validation for assessing these genes at the molecular level is being pursued, by referring to a study by Samad et al., (2020) [39] as an example. Notwithstanding the limitations, this review had provided new intuition into the dysregulation of lipid metabolism upon SARS-CoV-2 infection for further studies.

## Conclusions

From the results of this review, fatty acid and cholesterol homeostasis could be considered the main biological processes altered by SARS-CoV-2 infection. Dysregulation of these pathways would affect the pathogenicity of the virus, mainly for inflammation and prevention of viral replication. Thus, the importance of *PPARγ*, *APOBEC3G* and *IFITM3* upon viral infection could not be denied due to their involvement in pathways affecting viral pathogenicity, specifically in viral replication. Therefore, further studies on targeting these lipid metabolic pathways-associated genes were needed to identify potential biomarkers that could lead to the development of new therapeutic strategies to prevent viral replication and enhance the treatment of COVID-19.

### Electronic supplementary material

Below is the link to the electronic supplementary material.

**Supplementary Material 1: S1 Table.** Quality Assessment of Selected Studies

**Supplementary Material 2: S2 Table.** The significant functional annotations of DEGs related to lipid metabolism

**Supplementary Material 3: S3 Table.** The list of 213 DEGs involved in lipid metabolisms pathways analyzed using the STRING online database

**Supplementary Material 4: S4 Table.** The full list of significant functional annotations of all DEGs in each cluster

**Supplementary Material 5: S5 Table.** The details on the functions and terms related to candidate genes

**Supplementary Material 6: S6 Table.** Combined score of DEGs interactions

## Data Availability

The data that supports the findings of this study are available in the supplementary information of this article.

## Abbreviations

2019-nCoV: 2019 novel Coronavirus
ACE2: Angiotensin Converting Enzyme 2
*APOBEC3G*: Apolipoprotein B mRNA editing enzyme catalytic subunit 3G
BALF: Bronchoalveolar lavage fluid
BBID: Biological Biochemical Image Database
CITE-seq: Cellular Indexing of Transcriptomes and Epitopes by Sequencing
COVID-19: Coronavirus Disease 2019
DAVID: Database for Annotation, Visualization, and Integrated Discovery
DEG: Differentially Expressed Gene
FDR: False Discovery Rate
GO: Gene Ontology
HDL: High-density lipoprotein
*IFITM3*: Interferon-induced transmembrane protein 3
KEGG: Kyoto Encyclopedia of Genes and Genomes
LDL: Low-density lipoprotein
MCODE: Molecular Complex Detection
MeSH: Medical Subject Heading
NGS: Next-generation sequencing
PBMC: Peripheral blood mononuclear cell
*PPARγ*: Peroxisome proliferator-activated receptor gamma
PPI: Protein-Protein Interaction
PRISMA: Preferred Reporting Items for Systematic Reviews and Meta-Analyses
RAAS: Renin-Angiotensin-Aldosterone System
SARS-CoV-1: Severe Acute Respiratory Syndrome Coronavirus 1
SARS-CoV-2: Severe Acute Respiratory Syndrome Coronavirus 2
VAPA: VAMP Associated Protein A
